# Retrospective study of smartphone usage effects on tension-type headache prevalence in young adults

**DOI:** 10.6026/973206300213958

**Published:** 2025-10-31

**Authors:** Vishal Janarthanan, Jordan Ridhay Rasquinha, Shanmukha Koppolu, Manova Sam Shalin V.R, Balamurali K, Sr Vijaya Madhuri Devi Kunche, Shoraf Pascal

**Affiliations:** 1 Department of ICU, SRM Institutes for Medical Science, Vadapalani, Chennai, India; 2 Deaprtment of Cardiology, Manipal Hospital Millers Road, Bengaluru, Karnataka, India; 3 Department of Trauma and Orthopaedics, Barts NHS Trust, London, England, UK; 4 Department of Psychiatry, Madha Medical College Hospital and Research Institute, Kovur, Chennai, Tamil Nadu, India; 5 Department of Critical Care Medicine, JR, Dr. NTR University of Health Sciences, Vijayawada andhra Pradesh, India; 6 Department of Community Medicine, Madha Medical College and Research Institute, Kovur, Chennai, Tamil Nadu, India

**Keywords:** Tension-type headache, smartphone usage, screen time, young adults, posture

## Abstract

Tension-type headache (TTH) is rising among young adults, potentially linked to increasing smartphone use. This retrospective study investigated the association between smartphone usage and TTH among 120 participants aged 18-30 years. Individuals with screen time exceeding 4 hours/day reported significantly more frequent and severe headaches. Posture and type of smartphone activity also influenced headache intensity. Thus, the findings call for behavioral interventions to mitigate TTH risk.

## Background:

Smartphone a usage is becoming near universal across the globe, with the most accelerated rate of growth occurring among young adults who utilize smartphones for their education, employment and socialization [1]. Recent international survey data indicate the average daily screen time of adults aged 18-30 often surpasses 4 to 6 hours a day, thus recording behavioral changes and shifts about digital overreliance on smartphones [2]. While smartphones are of significant utility, over-reliance on the smartphone features has been highlighted as a causative factor in a range of physical and neurological repercussions, including visual fixation, poor ergonomics and forward neck posture through prolonged periods of use [3]. Tension-type headache (TTH) represents the most prevalent primary headache disorder globally; with lifetime prevalence rates as high as 38% in the population during life and is particularly prevalent in young adults [4]. TTH has a defined clinical presentation characterized by a dull, bilateral, tightening or pressing sensation of pain, which is most commonly associated in the literature with psychological, physical stress and musculoskeletal changes and has increased risk of impaired cognition and decreased levels of productivity/loss of productivity, harmful physical outcomes, chronic pain, significant loss and diminished overall quality of life [5]. Much of the research has investigated the general and broad associations of digital screen exposure and associated health issues including eye strain, sleep issues, neck pain and etc. but little investigation has focused on the meaningful contribution of the smartphone by exploring smartphone use and its relationship with TTH and in contributing to the onset and exacerbation of TTH [6]. Patterns of behavior, including too much recreational screen time each day using a smartphone, constant texting or gaming with friends, poor sitting posture and lack of breaks have been named possible risk factors of TTH, but available scientific evidence is still sparse and inconsistent [7]. Also, young adults, as a population, are a crowd likely a particularly susceptible crowd because of greater academic, occupational and social pressures than youth possess. The existing pressures faced by young adults may, in combination with additional strain associated with smartphone-use, contribute to increased discomfort and regard screens as an activity which is positively reinforced when socially exchanged with friends [8]. Therefore, it is of interest to investigate the impact of smartphone usage patterns on the prevalence and severity of tension-type headache among young adults.

## Materials and Methods:

This retrospective study was conducted on 120 young adults between the ages of 18 and 30, recruited from outpatient clinics over one year. Participants were grouped into low (1-2 hours), moderate (3-4 hours) and high (5+ hours) daily smartphone usage categories. Data sources included clinical records, structured questionnaires and one-year retrospective headache diaries. Participants provided details about their screen time, headache frequency, severity, posture during usage and specific smartphone activities (e.g., gaming, social media, work). Lifestyle factors such as sleep duration, physical activity and stress levels were also documented. Statistical analysis was conducted using SPSS, employing both descriptive statistics and inferential methods (chi-square test, ANOVA) to evaluate the correlations between smartphone use and TTH patterns.

## Results:

The research determined a significant link between increased smartphone screen time and the prevalence, frequency and severity of tension-type headaches (TTH). The majority of participants were classified as moderate users (37.5%), with high users accounting for 29.2%. High users reported frequent tension-type headache episodes (71.4%), which was much more than moderate users (44.4%) and low users (25%) and severe headaches in general was more common in high users (28.6%). Headache severity was exaggerated by poor posture; 25% of participants with poor posture reported severe TTH compared to only 6.7% TTH among those with good posture. Minimal specific smartphone activities showed a relationship with headache severity, although gaming and long durations of work-related use did stand out as longer durations of these activities were associated with more severe headaches. Sleep patterns also affected headaches, particularly sleep duration, where participants sleeping less than six hours reported the highest frequency of TTH (70%), as opposed to longer durations of sleep which had lower frequencies. Participants who were sedentary reported more frequent headaches (71.4%) than moderate activity (37.5%) and participants who exercised regularly (22.2%). High screen time compounded with poor posture increased headache frequency, as reported by 61.1% of participants. Regularly taking away from screen time reduced frequency and severity and no participants reported severe TTH who took screen time breaks every 30 minutes. The multiple regression analysis verified that long screen time, bad posture and short sleep duration were significant independent predictors of TTH, while physical activity was not statistically significant. Overall, these findings confirm the multifactorial nature of TTH and point to the role of behaviors and lifestyle factors that can, in concert, exacerbate and relieve symptoms.

Table 1 (see PDF) presents the distribution of participants by daily smartphone usage, with the largest group being moderate users (37.5%), followed by low (33.3%) and high users (29.2%). Table 2 (see PDF) shows that frequent TTH episodes were most common in high users (71.4%), compared to 44.4% of moderate users and 25% of low users. Table 3 (see PDF) demonstrates that severe headaches were more prevalent among high users (28.6%) than moderate (11.1%) and low users (5%). Table 4 (see PDF) presents the effect of posture, showing that poor posture was linked to more severe headaches (25%) compared to good posture (6.7%). Table 5 (see PDF) shows that gaming produced the highest proportion of severe headaches (33.3%), followed by work-related use (25%) and social media (16.7%). Table 6 (see PDF) demonstrates that participants sleeping less than six hours reported the highest frequency of headaches (70%) versus 37.3% with 6-8 hours and 26.3% with >8 hours of sleep. Table 7 (see PDF) presents the role of physical activity, showing frequent headaches were most common in sedentary individuals (71.4%), compared to 37.5% with moderate activity and 22.2% with regular exercise. Table 8 (see PDF) shows that combined high smartphone use and poor posture dramatically increased frequent headaches (61.1%) compared to high use with good posture (31.3%). Table 9 (see PDF) demonstrates that regular screen breaks reduced headache severity, with no severe cases in those taking breaks every 30 minutes compared to 31.3% in those rarely taking breaks. Table 10 (see PDF) presents the multivariate regression analysis, identifying screen time, poor posture and short sleep as significant independent predictors of TTH, while physical activity was not.

## Discussion:

This study reinforces the growing concern that excessive smartphone use among young adults contributes to the increased prevalence and severity of tension-type headaches [9]. Those using smartphones for more than 4 hours a day, especially in poor postural positions, experienced significantly more frequent and severe headaches [10]. Notably, specific activities such as extended engagement with social media or work-related use further amplified the symptom severity. These findings are consistent with prior research suggesting a link between prolonged digital exposure and musculoskeletal strain [11]. In addition to screen time, poor posture and associated sedentary behavior emerged as crucial factors [12]. Slouched or forward-head posture, often adopted unconsciously during phone use, can place strain on the cervical spine and occipital muscles, triggering or intensifying TTH episodes [13]. Furthermore, the psychological stress associated with constant digital connectivity and disrupted sleep patterns may also contribute to headache chronicity. This multidimensional causality highlights the need for a holistic approach to prevention. Educational interventions focusing on posture correction, digital hygiene and screen-time limitations should be prioritized, especially in university and workplace settings [14]. Although the retrospective design limits causal inference, the strength of the associations found here underscores the importance of future longitudinal studies to establish causality and evaluate intervention efficacy [15].

## Conclusion:

Increased smartphone use, particularly beyond 4 hours per day, is associated with more frequent and severe TTH in young adults. Poor posture and activity type further compound headache severity. Promoting ergonomic awareness and limiting screen time may help reduce TTH burden in this population.
